# Application of the RE-AIM Framework for the Pediatric Mild Traumatic Brain Injury Evaluation and Management Intervention: A Study Protocol for Program Evaluation

**DOI:** 10.3389/fpubh.2021.740238

**Published:** 2022-02-17

**Authors:** Paula W. Tucker, Rachel Bull, Alex Hall, Tim P. Moran, Shabnam Jain, Usha Sathian, Harold K. Simon, Gerard A. Gioia, Jonathan J. Ratcliff, David W. Wright

**Affiliations:** ^1^Nell Hodgson Woodruff School of Nursing, Emory University, Atlanta, GA, United States; ^2^Department of Emergency Medicine, Emory University School of Medicine, Atlanta, GA, United States; ^3^Departments of Pediatrics and Emergency Medicine, Emory University School of Medicine and Children's Healthcare of Atlanta, Atlanta, GA, United States; ^4^Urgent Care and Community Care Services, Children's Healthcare of Atlanta: Department of Pediatrics, Emory University School of Medicine, Atlanta, GA, United States; ^5^Division of Pediatric Neuropsychology, Children's National Hospital, Rockville, MD, United States

**Keywords:** RE-AIM, mix-methods, program evaluation, mild traumatic brain injury, concussion, pediatric, intervention

## Abstract

**Background:**

Children who experience a mild Traumatic Brain Injury (mTBI) may encounter cognitive and behavioral changes that often negatively impact school performance. Communication linkages between the various healthcare systems and school systems are rarely well-coordinated, placing children with an mTBI at risk for prolonged recovery, adverse impact on learning, and mTBI re-exposure. The objective of this study is to rigorously appraise the pediatric Mild Traumatic Brain Injury Evaluation and Management *(TEaM)* Intervention that was designed to enhance diagnosis and management of pediatric mTBI through enhanced patient discharge instructions and communication linkages between school and primary care providers.

**Methods:**

This is a combined randomized and 2 × 2 quasi-experimental study design with educational and technology interventions occurring at the clinician level with patient and school outcomes as key endpoints. The RE-AIM (Reach, Effectiveness, Adoption, Implementation, Maintenance) framework will be utilized as a mix methods approach to appraise a multi-disciplinary, multi-setting intervention with the intent of improving outcomes for children who have experienced mTBI.

**Discussion:**

Utilization of the RE-AIM framework complemented with qualitative inquiry is suitable for evaluating effectiveness of the *TEaM* Intervention with the aim of emphasizing priorities regarding pediatric mTBI. This program evaluation has the potential to support the knowledge needed to critically appraise the impact of mTBI recovery interventions across multiple settings, enabling uptake of the best-available evidence within clinical practice.

## Introduction

Mild Traumatic Brain Injury (mTBI) is a considerable public health problem that is responsible for more than 70% of traumatic brain injuries in the United States (1, 2). Between 2004 and 2013, head injury among children up to 17 years of age accounted for 1.6 million outpatient (68%) and Emergency Department (ED) (32%) visits (3). In 2014 alone, TBI-associated ED visits, hospitalizations, and deaths comprised more than 837,000 children (4).

Children who sustain an mTBI may be seen in multiple healthcare settings across their recovery, such as primary care, urgent care, specialty clinics, ED, and inpatient settings (5, 6), while most of the recovery phase occurs in the home and school settings (5). Unfortunately, inconsistency in management approaches and treatment recommendations exist across the various healthcare settings (5, 7). Adding to this complexity is the reality that communication linkages between the healthcare systems and the schools are frail. As a result, this lack of communication and care coordination increases the risk of re-exposure to mTBI, less effective management in healthcare settings, and delayed recovery (5).

Current needs and opportunities for improvement exist in the management of mTBI in children, explicitly traversing across the care continuum from emergency and urgent care to primary care settings and then to the schools where the child spends a significant amount of their day. Therefore, we view the ecology of mTBI services as akin to a neighborhood, with various elements that must work together to be a successful community.

A growing number of studies offer insight into the consequences of mTBI (8–13). Children who sustain a TBI, including mTBI, may encounter cognitive and behavioral changes that can negatively influence school performance (13–16). These changes may even influence the quality of life in childhood and adult years (8, 17, 18). For most children who experience symptoms related to mTBI, recovery typically occurs between 1 and 6 weeks (19–21); however, symptoms can persist up to 3 months or longer following injury (10, 17, 21, 22).

In 2018, the Centers for Disease Control and Prevention (CDC) and the Pediatric Mild Traumatic Brain Injury Workgroup published the first evidence-based guidelines encompassing 19 best practice recommendations regarding identification, prognosis, and management of mTBI among children including recommendations for return to school (23). These recommendations were based on the findings of a recent systematic review (24). Specific intervention strategies and recommendations have also been proposed to promote improved academic and healthcare outcomes for students with mTBI (5, 7, 25–28). Despite a better understanding of pediatric mTBI, there remains heterogeneity in diagnosing and managing the condition. This variation may be attributed to a lack of standardization and widespread adoption of evidence-based clinical guidelines, including guidance on return to school (29–34). Therefore, a systematic approach and the integration of clinical decision support tools to facilitate knowledge translation for advancing diagnosis and management of pediatric mTBI are promising strategies that may improve clinician training and decision making in the care delivery process for this vulnerable population (31–33). To ensure maximum effectiveness, the diagnosis and individualized management strategies from the primary healthcare provider must be communicated to the child, parent, and school personnel through a standardized, consistent pathway (25, 28).

In response to the pediatric mTBI public health concern, the CDC released a notice of funding opportunity to improve pediatric mTBI outcomes through healthcare provider training, clinical decision support, and discharge instructions. The study funded through this notice, the mild Traumatic Brain Injury Evaluation and Management (*TEaM*) Intervention, is a multi-faceted intervention that includes sophisticated decision support in pediatric primary care (PC), pediatric urgent care (UC), and pediatric EDs. The study's focus is directed toward improved diagnosis and management of pediatric mTBI, including patient discharge education and enhanced communication among school and healthcare providers. The purpose of this manuscript is to apply the Reach, Effectiveness, Adoption, Implementation, and Maintenance (RE-AIM) framework to critically evaluate the planned multi-disciplinary, multi-setting intervention designed to improve outcomes for children who have experienced mTBI (35–37). In addition to overall program evaluation, the RE-AIM Qualitative Evaluation for Systematic Translation (RE-AIM QuEST) developed by Forman et al. will be used to inform qualitative data collection for the planning and formative evaluation of the *TEaM* Intervention (38). The focus of this paper is to define the feasibility, sustainability, and capability of scaling up implementation of the *planned TEaM Intervention*, including implementation of the mTBI training program across clinical settings.

The RE-AIM model was first developed in 1999 by Glasgow et al. (35) as an evaluative framework to assess the public health influence of health promotion initiatives with an emphasis on gathering information regarding their Reach, Effectiveness, Adoption, Implementation, and Maintenance (35). It assists researchers in the planning, evaluating, and reporting of healthcare intervention effectiveness (35–37). The RE-AIM model considers the long-term impact of an intervention within real-world settings, using a multi-level (individual, organizational) approach (35), and is adaptable to effectiveness studies such as randomized control trials (39, 40), time-series, as well as quasi-experimental designs (41, 42). In this model, Reach is the number, percentage, and representativeness of individuals agreeing to participate in an intervention, including the rationale for participation and non-participation. Effectiveness is the influence of the intervention on significant outcomes such as quality of life, economic, and potential negative or positive effects. Adoption is the number, percentage, and representation of settings and participants agreeing to deliver the initiative, including the rationale for adoption and non-adoption. Implementation is the consistency to which various intervention components are conducted as proposed, including intervention modifications and implementation strategies. Lastly, Maintenance is the degree to which the intervention becomes part of routine organizational processes, and the intervention's long-term impacts on individual-level outcomes (36, 37). Emphasis on external validity originates from the Reach and Adoption dimensions, and internal validity originates from Effectiveness and Implementation dimensions of the model (43).

As a mix-methods (quantitative, qualitative) approach to evaluate the planned *TEaM* Intervention, this manuscript will attempt to answer the following questions directed by both the RE-AIM (36, 44) and RE-AIM QuEST (38) frameworks: Is the *TEaM* Intervention reaching healthcare providers who diagnose and manage children with mTBI? How effective is the *TEaM* Intervention on mTBI-related outcomes, including adverse effects on academic performance and health-related quality of life among school-aged children with concussions? To what extent did healthcare settings that were proposed to deliver the *TEaM* Intervention actually participate? To what degree was the *TEaM* Intervention consistently deployed across study sites? To what degree and time point did the *TEaM* Intervention become standard of practice and sustain effectiveness? See Table 1 for *TEaM* Evaluation Data Collection Plan.

**Table 1:** *TEaM* evaluation data collection plan.

**RE-AIM dimensions**	**Quantitative**	**Qualitative**
**REACH**		
**Numerator** # of HCPs who participate in the study (intervention/control) **Denominator** Total # of HCPs eligible to consent (intervention/control/non-participation) across settings (ED, UC, PC) **Data sources** ED personnel data eMR surveys/questionnaires semi-structured interviews focus groups provider training records	**Inclusion criteria** # of HCPs who consent to the study **Exclusion criteria** # of HCPs unwilling or ineligible to consent **Representativeness among HCPs** Basic demographics Similarities/differences: participation/non-participation, control/intervention groups	Barriers/facilitators to HCP recruitment, reasons for participation and non-participation **Formative evaluation (study phase 2-provider recruitment)** Assess barriers to recruitment and how barriers were addressed Assess provider knowledge and skills in mTBI diagnosis and management Obtain provider feedback regarding mTBI training modules
**EFFECTIVENESS**		
**Validated study instruments** CDC ACE Tools (45, 46) CLASS-3 (parent/student report) (14, 47) PCSI-2/PCEI (48, 49) PEDS QL4 (50, 51) **Data sources** eMR surveys/questionnaires telephone interviews	**Definition** Comparative change/improvements in mTBI clinical outcome measures over time (1, 2, 4, and 12 weeks) **mTBI Clinical Outcome Measures** **Primary outcomes** **academic problems/decline after return to school** CLASS-3 (parent/student report) (14, 47) (weeks 1, 2, 4) **Secondary outcomes mTBI symptoms post injury** PCSI-2 (48) (weeks 1, 2, 4, and 12) **anxiety/mood changes post injury AND after return to school** PCSI-2/PCEI (48, 49) (week 1) PCSI-2 emotion scale (48) PCEI emotional control scale (49) CLASS-3 school stresses (14, 47) after return to school (weeks 2, 4, and 12) **PEDS QL4** quality of life after return to school (50, 51) (weeks 4 and 12) **Time to Recovery** (45, 46)	Circumstances and processes influencing *TEaM* Intervention effectiveness Differences in patient-level outcomes between intervention vs. control groups across study sites (ED, UC, PCP)
**ADOPTION**		
**(ED, UC, PC settings)** **Numerator** # of settings that adopted *TEaM* Intervention components **Denominator** Total # of settings approached **Data sources** eMR training records semi-structured interviews site visits observations	**Definition** #/% of *settings willing to* adopt *TEaM* Intervention components **Representativeness of settings** Basic demographics Similarities/differences among settings: ED, UC, PC (baseline initiatives vs. intervention components), adoption vs. non-adoption	Understand contextual factors influencing adoption of *TEaM* Intervention components Identify barriers and facilitators to adoption and non-adoption across study sites **Formative evaluation** **(study phase 1-building a foundation)** Assess stakeholder perceptions and feedback regarding educational and CDS tools Identify anticipated barriers/facilitators to adoption of *TEaM* Intervention components across delivery systems (ED, UC, PC)
**IMPLEMENTATION**		
**Numerator** # of individual *TEaM* intervention components implemented **Denominator** Total # of *TEaM* intervention components **Data sources** eMR informal interviews focus groups among providers site visits observations	**Definition** Degree of *TEaM* intervention components consistently implemented across sites **Setting/*****TEaM*** **intervention components** **ED and UC settings** 1. Provider Training Modules 2. eMR Triage screening/provider alert 3. eMR Concussion Smart Form Template (documentation, decision support, management pearls) 4. Triggered by eMR Concussion Smart Form Template → CDC evidence-based discharge instruction →Communication Linkage Letters - Return to School with suggested accommodations - Return to Primary Care with ED findings **Primary care settings** 1. Concussion Academy Skill Training (CAST) and Training Modules 2. eMR Concussion Smart Form Template (documentation, decision support, management pearls) 3. CDC ACE tool (45, 46) 4. CDC ACE care plan/education (45, 46) 5. Linkage letters - Return to School with STAMP suggested accommodations and supports	Understand variations of implementing the *TEaM* Intervention components Approach to adapting each component of the *TEaM* Intervention across delivery systems Implementation barriers and facilitators of the *TEaM* Intervention including contextual factors and operational practices and how they were addressed
**MAINTENANCE**		
**data sources** eMR informal interviews site visits	**Definition** Utilization of *TEaM* intervention components among HCPs 6 months following the study period	Identification and engagement of champions across delivery systems Understand barriers to sustainability of the *TEaM* Intervention across study sites
		**Formative evaluation** **(completion of study phase 3-subject enrollment and pre-decision support)/study phase 6 (study wrap up)** Evaluate provider experiences, perceptions, barriers and facilitators regarding *TEaM* Intervention components Asses contextual factors underlying facilitators and barriers to implementation of *TEaM* Intervention components

## Methods

### Study Design Overview

The *TEaM* study is a combined randomized and 2 × 2 quasi-experimental study design with training and technology interventions occurring at the provider level involving ED, UC, PC healthcare providers (HCPs) across the Children's Healthcare of Atlanta system, with patient and school outcomes as key endpoints. At the HCP level, this study is randomized, and at the patient level, this study is a quasi-experimental with a pre and post design to facilitate analysis of the impact of our Electronic Medical Record (eMR) support tool on children's health outcomes.

### Participants/Setting

Participants in this study include HCPs in ED, UC, and PC settings affiliated with Children's Healthcare of Atlanta. HCPs will include Physicians and Advanced Practice Providers (nurse practitioners and physician assistants). HCP level randomization will be stratified by practice site to ensure that the distribution of control and intervention providers is approximately equal within sites. Providers will be sent an electronic informed consent form (eICF) using Research Electronic Data Capture (REDCap™) tools (52) for consent to participate.

Children ages 5–18 years old who screen positive and/or are diagnosed with mTBI/concussion will be recruited following discharge from their index injury visit, which may have occurred in either ED, UC or PC settings. Regular reports of children who screen positive through the decision support tool will be sent to the research team throughout the 32-month study period. After confirmation of an evaluation by a participating provider and either a positive screen or diagnosis of mTBI/concussion, an initial phone call will be made to the parent.

Upon the first contact, the parent will be given a very brief explanation of the study at which point, an option to opt-out will be offered. If they choose to opt-in, a simple validated symptom questionnaire, the CDC Acute Concussion Evaluation (ACE) tool (45) will be used to ensure that the child meets eligibility requirements. At this point, if the child is eligible, parental verbal consent will be obtained in order to proceed with further explanation of the study. A total of 532 consecutive children (see section Data Analyses) with mTBI meeting inclusion/exclusion criteria will be recruited. The goal is to maintain a lost to follow up (LTF) rate of <20% for a total sample of 425. Enrolled children will be treated for up to 6 months post-injury to assess the impact of the *TEaM* Intervention on pediatric mTBI-related outcomes.

### *TEaM* Intervention

The *TEaM* Intervention was developed by the investigators through rigorous evaluation of the literature, incorporation of the CDC guidelines (23, 45, 46), and assimilation of best practices currently adopted by national experts (31, 45). The *TEaM* Intervention will be adapted for this study in collaboration with stakeholders from pediatric neuropsychology, emergency medicine, neurosurgery, sports medicine, primary care, ambulatory care, clinical informatics, nursing, school linkage experts, educators, school health personnel, as well as members of the CDC Pediatric Mild Traumatic Brain Injury Guideline Workgroup (23). The intervention consists of provider training in mTBI evaluation, care coordination and management protocols, eMR decision support system, and linkage materials. The eMR will be leveraged to refine the provider training portion of the intervention to improve screening, management, and linkage throughout the clinical care continuum with the goal of producing an effective and scalable intervention aimed at reducing mTBI-related school problems/performance, improving patient symptom recovery, and reducing post-injury complications. The *TEaM* Intervention is designed to enhance adoption and standardize best practices; the study tools are designed to fully function without interfering with a system that already has a high-functioning mTBI program. Most importantly, this intervention enables potential generalizability across various clinical settings; thereby, improving the translation and adoption of evidence-based management for patients, caregivers, providers, and school personnel to promote recovery following pediatric mTBI.

## Data Collection

### Reach (Provider Level): Is the *TEaM* Intervention Reaching HCPs Diagnosing and Managing Children With mTBI?

We will determine how many and what proportion of HCPs receive *TEaM* Intervention training within each proposed site (ED, UC, PC) (36). The numerator is defined as the number of HCPs who agree to participate and receive the *TEaM* Intervention training. The denominator is defined as the total number of potential HCPs who are eligible for the study across each study site (53). We will consider representativeness by identifying similarities and/or differences between HCPs (intervention vs. control group and participation vs. non-participation). Comparisons among the control and intervention groups regarding compliance to the protocol and basic demographic characteristics will also be assessed (36). Qualitative inquiry will complement our data collection on Reach. Our objective is to understand how HCPs perceive the *TEaM* Intervention training, including barriers and facilitators to recruitment, participation, and non-participation across sites (38). Data sources will include eMR, provider questionnaires, semi-structured interviews, focus groups, and attendance records from provider training.

### Effectiveness/Efficacy (Patient Level): How Effective Is the *TEaM* Intervention at Improving mTBI-Related Outcomes, Including Academic Performance and Health-Related Quality of Life Among School-Aged Children With Concussions?

The primary outcome of the *TEaM* Intervention is to determine if implementation of an evidence-based/best practice mTBI intervention with multi-setting communication linkages will decrease mTBI-related complications among school-aged children, a universal endpoint. Effectiveness of the intervention for the intervention group vs. controls (38) will be examined on important patient level outcomes utilizing validated instruments. The primary outcome is degree of academic problems as reported by the parent and student on the Concussion Learning Assessment and School Survey, 3rd Ed (**CLASS-3**) (14, 47). Secondary outcomes will be the emotional responses of the students as measured by the Emotional Symptom scale of the PostConcussion Symptom Inventory-2 (**PCSI-2**) (48), the Emotional Control scale of the PostConcussion Executive Inventory (**PCEI**) (49), and the School Stresses scale of the CLASS-3. General well-being will be assessed by the Pediatric Quality of Life Inventory (**PEDS QL4**) (50, 51). Each of these measures will be administered at 1, 2, 4, and 12 weeks. Finally, time to recovery (45, 46) will be assessed for each study group. Attrition rates of patients will also be determined. Further data collection will include qualitative inquiry on effectiveness. Our objective is to determine the circumstances and processes that resulted in *TEaM* Intervention effectiveness and the differences in patient-level outcome measures across study sites (38). Data sources will include eMR, surveys, telephone interviews with patients and parents.

### Adoption (Organizational Level): To What Extent Did Healthcare Settings That Were Proposed to Deliver the *TEaM* Intervention Actually Participate?

We will assess adoption of the *TEaM* Intervention at the organizational level. We will determine the number and proportion of study sites (ED, UC, PC) that adopt the components of the *TEaM* Intervention (increased utilization of best practices in diagnosis, follow-up by patients and their parents or guardians, and number of Return to School/Primary Care Letters provided), and how many did not adopt the intervention (36). The numerator is characterize as the number of settings that adopted individual *TEaM* Intervention components. The denominator is defined as the total number of settings approached (53). We will also consider the representativeness of each study site (36) by assessing similarities or differences between current practices (baseline programs and initiatives) against *TEaM* Intervention components (use of validated symptom inventories, appropriate exam documentation, provision of CDC or institutional discharge instructions, return to school/primary care letters, appropriate use of diagnostic tools (e.g., computed tomography when indicated), identification and referral for children with persistent post-concussion symptoms). Our objective is to understand contextual factors that influence adoption, including the barriers and facilitators to adoption and non-adoption across study sites (38, 44). Data sources will include eMR, semi-structured interviews, site visits, and observations.

### Implementation (Provider Level): To What Degree Was the *TEaM* Intervention Consistently Deployed Across Study Sites?

Individual provider-level evaluation will determine the percentage of provider utilization of the *TEaM* Intervention components across study sites and the use of evidence-based practices for diagnosing and managing mTBI. The provider utilization rate of *TEaM* Intervention components will be determined by a numerator (# of individual *TEaM* Intervention components consistently implemented) and a denominator (Total # of *TEaM* Intervention components). We will also evaluate the extent of communication linkages between providers (ED, UC, and PC), patients, parents, and school personnel (25). To evaluate implementation components of the *TEaM* Intervention (provider training, eMR-based screening, notification system and decision-support, patient discharge instructions, post-mTBI management), we will determine the consistency of implementation across each study site through provider utilization rate of individual intervention components, the length of time, cost, and resource utilization associated with the implementation of the intervention (36). Strategies to improve implementation of the *TEaM* Intervention will include provider training, technical assistance, and ongoing feedback surrounding the utilization of the *TEaM* Intervention components (36). Our goal is to understand the variations (similarities and differences) of implementing the *TEaM* Intervention and the approach to adapting each component of the *TEaM* Intervention across delivery systems (38, 44). Implementation barriers and facilitators of the *TEaM* Intervention will be determined, including contextual factors and operational practices triggering the barriers to implementation and how they were addressed (38). Data sources will include eMR, informal interviews, focus groups among providers, site visits and observations.

### Maintenance (Provider Level): To What Degree and Time Point Did the *TEaM* Intervention Become Standard of Practice and Sustain Effectiveness?

Individual provider-level maintenance will be determined by utilization of the *TEaM* Intervention components and evidence-based practices in diagnosing and managing mTBI among HCPs 6 months following the end of the study period (36). To maintain the sustainability of the *TEaM* Intervention across sites and facilitate communication between HCPs and school personnel, engagement of stakeholders across delivery systems will be reinforced on mTBI best practices to promote patient recovery (25). Qualitative inquiry will complement data collection on individual level maintenance by understanding the barriers to sustainability of the *TEaM* Intervention across study sites (38). We will explore strategies to facilitate appropriate work-flow and practice changes and seek new sources of funding opportunities to help promote sustainability and scalability of the *TEaM* Intervention. Data sources will include site visits and interviews with site specific provider champions following the study period.

### Formative Evaluation

**Figure 1:**
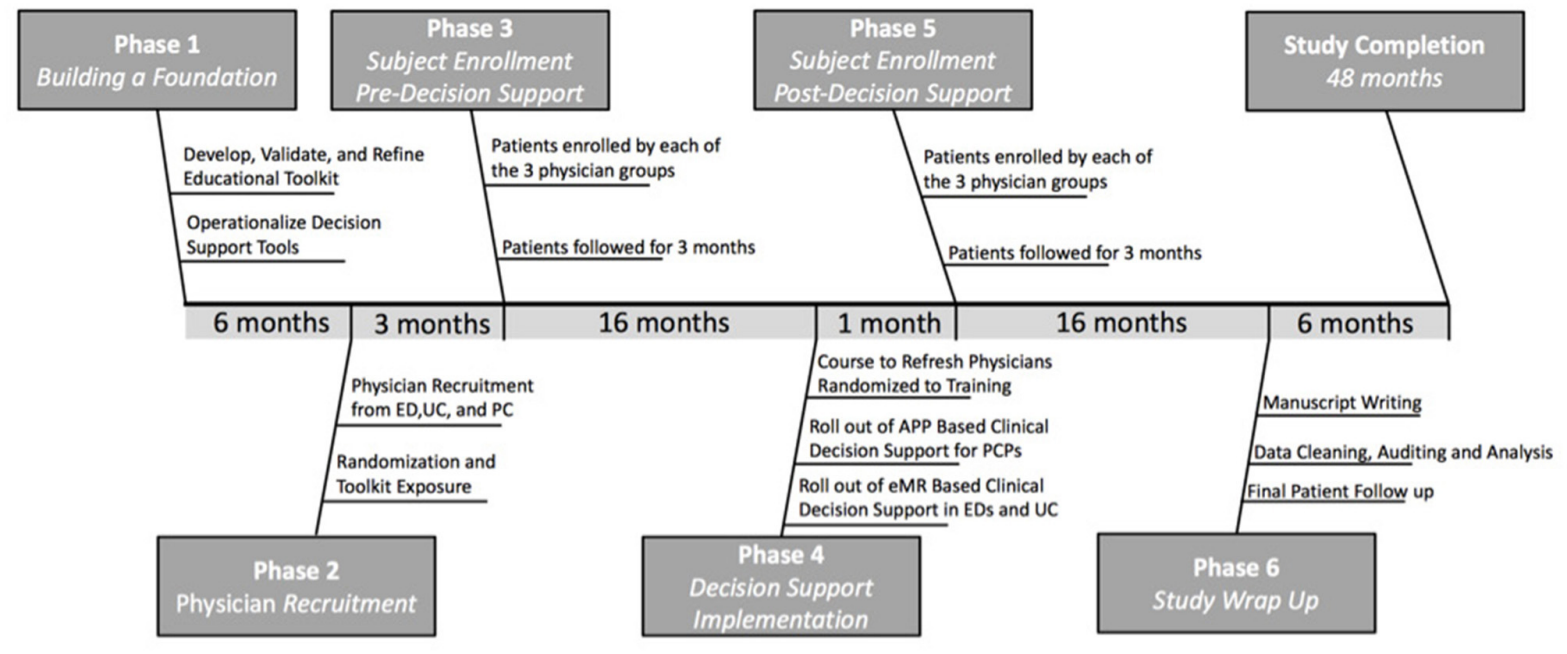
*TEaM* study timeline.

Formative evaluation of the *TEaM* Intervention will be guided by the RE-AIM QuEST Framework (38) as an expansion of RE-AIM (36) to inform qualitative data collection throughout each phase of the study period (Figure 1). To facilitate Adoption and Implementation, during study phase 1 (*Building a Foundation*), we will identify and assess stakeholder (providers, hospital administrators, IT system personnel) perceptions and feedback regarding the training and CDS tools, and anticipated implementation barriers and facilitators surrounding utilization of the *TEaM* Intervention components across delivery systems (ED, UC, PC). To facilitate Reach, during study phase 2 (*Provider Recruitment*), we will assess any barriers to recruitment and how these barriers were addressed (38). We will also assess provider knowledge and skills relating to mTBI diagnosis and management during recruitment and obtain provider feedback regarding the mTBI educational training modules. To facilitate Maintenance, at the completion of study Phase 3 (*Subject Enrollment and Pre-Decision Support*) and during study Phase 6 (*Study Wrap Up*) of the *TEaM* Intervention, we will evaluate provider experiences, perceptions, barriers, and facilitators regarding *TEaM* Intervention components across delivery systems. Reasons for adaptations to the *TEaM* Intervention components, and assessment of contextual factors underlying facilitators and barriers to implementation of the *TEaM* Intervention across delivery systems will be completed (38). Finally, we will evaluate provider, patient, family and school personnel experiences, perceptions, barriers, and facilitators regarding communication linkages across delivery systems. Qualitative data collection will include focus groups, formal meetings, semi-structured interviews, and surveys.

### Data Analyses

#### Sample Size

Sample size calculations were conducted with the aim of achieving 80% power for the primary outcome. Computations were based on previous research using the Concussion Learning Assessment and School Survey, 3rd Ed. (CLASS-3) which reported on the mean, variability, and intercorrelations for metrics derived from the CLASS (47). The sample size was calculated for a 1:1 treatment allocation scheme with 80% power to detect an overall 20% difference in the mean of the CLASS-3 Academic Problems Scale at the two-sided α level of 0.05 (corresponding to a Cohen's ≈ 0.15). With these parameters, we will randomize 532 patients after assuming a loss-to-follow-up rate of 20%. Assuming minimal loss-to-follow-up, power is expected to be ~88%.

#### Descriptive Statistics

Frequencies and percentages will be used to describe categorical variables. Means, standard deviations, medians, and interquartile ranges will be used to describe continuous variables. The reliability of variables obtained via chart review will be evaluated using intraclass correlations, Cohen's κ, or Fleiss' κ depending on the characteristics of the variable.

#### Primary Outcome—CLASS-3 Academic Problems Scale

Analyses will be conducted under the intention-to-treat principle (ITT). Scores on the CLASS-3 Academic Problems scale (42 pt. scale) will be compared between intervention and control patients using a mixed-effects/multilevel linear regression. Experimental group and Timepoint (weeks 1, 2, and 4) will be included as independent variables. The mixed-effects model will be used in order to account for clustering within the data (e.g., clustering within provider, patient, etc.). When possible, the intercept and intervention slope will be included as random effects to evaluate the variability between providers and sites. Alternatives will be considered if model assumptions are not met or if the model fails to estimate properly.

#### Secondary Outcomes—(PCSI Emotion Scale, PCEI Emotional Control Scale, CLASS-3 School Stresses Scale, PEDS QL4, Time to Recovery)

For continuous variables, the analyses will be the same as above: either a mixed-effects/multilevel linear regression. For other variables, the distribution family and link function will be determined by the characteristics of the data.

#### Study Covariates

Demographics (age, sex, race), premorbid conditions (migraines and other headaches, depression, ADD/ADHD, other learning disorders, and other psychiatric illnesses), prior mTBI history and duration of symptoms, baseline academic performance, provider type (e.g., APP/Physician) and specialty, initial number of mTBI symptoms, and mechanism of injury.

#### Subgroup Analyses

The following planned subgroup analyses will be conducted in order to assess for heterogeneity of treatment effects: (1) Patients seen by resident physicians will be compared with those not seen by a resident, (2) patients treated by providers in compliance with the protocol will be compared to those who were not, (3) patients who received school accommodations will be compared with those who did not, (4) the effectiveness of the interventions will be compared across provider type and specialty.

#### Missing Data

In the event of substantial missing data, data will be imputed using a multiple imputation procedure. The type of procedure (e.g., fully conditional specification, hot-decking, etc.) and the number of imputed data sets will be determined by the amount of missing data and the pattern of missingness. Efforts will be made to minimize loss-to-follow up; however, in the event that a patient's primary outcome (CLASS-3 score at the 1, 2, and 4-week follow ups) is unobtainable, we will (1) assume the worst and assign an “unfavorable” outcome for that patient in order to conduct the ITT analysis and (2) assume the best and assign a “favorable” outcome for that patient as a sensitivity analysis for the ITT analysis. These analyses will describe the boundary conditions for the models described above.

#### Multiple Testing

To the extent possible, we will track the number of analyses to report the false discovery rate, that is, the expected proportion of false positives. We will test the primary outcome variable at an α level of 0.05; for secondary outcomes, the false discovery rate will be formally controlled.

## Discussion

### Significance

The CDC has informative, evidence-based resources and existing instruments to support healthcare providers in the evaluation, management, and treatment of pediatric mTBI (https://www.cdc.gov/headsup/providers/index.html) (54). Although these resources are made available to all stakeholders and clinicians, the dissemination and implementation of intervention programs has not been rigorously evaluated in real-world settings, e.g., utilization and adoption across healthcare delivery systems (ED, UC, and PC). This study targets the public health concern and translational research gap in pediatric mTBI. We will use RE-AIM as a pragmatic model (44) to assist us in evaluating the implementation and effectiveness of a multi-component clinical intervention using clinician training, electronic clinical decision support, customized discharge instructions, and communication linkage tools to ultimately improve personalized diagnosis, management, and treatment of pediatric mTBI established from high-quality evidence.

### Public Health Impact

The *TEaM* Intervention's public health impact will be determined by healthcare provider reach and efficacy on the individual level and the implementation and adoption on the organizational level (35, 55, 56). We seek to carefully evaluate any translational and dissemination issues related to the application of the *TEaM* Intervention within real-world settings (43, 56), and believe this evaluation will provide further knowledge and inquiry for future studies, updates to evidence-based guideline recommendations and dissemination materials. Furthermore, evaluation of this intervention utilizing the RE-AIM framework (35, 55) is an appropriate method in determining the effectiveness and generalizability of the *TEaM* Intervention, providing the knowledge needed to inform our stakeholders (patients, parents, school personnel, providers and grant funders), and facilitate the translation of research into the clinical setting, thereby improving practice change, program sustainability and outcomes for children with mTBI.

### Contributions to the Field Statement

This study evaluates the impact of an evidence-based, multi-component pediatric mTBI intervention across multiple pediatric healthcare settings leveraging the eMR to facilitate clinical decision support and improve communication linkages since the release of the new CDC pediatric mTBI guidelines. Utilization of the RE-AIM framework (36) complemented with qualitative inquiry (RE-AIM QuEST) (38) to evaluate the impact of *TEaM* Intervention across multiple healthcare settings is a pragmatic approach in facilitating the translation of research into clinical practice.

**Figure 2:**
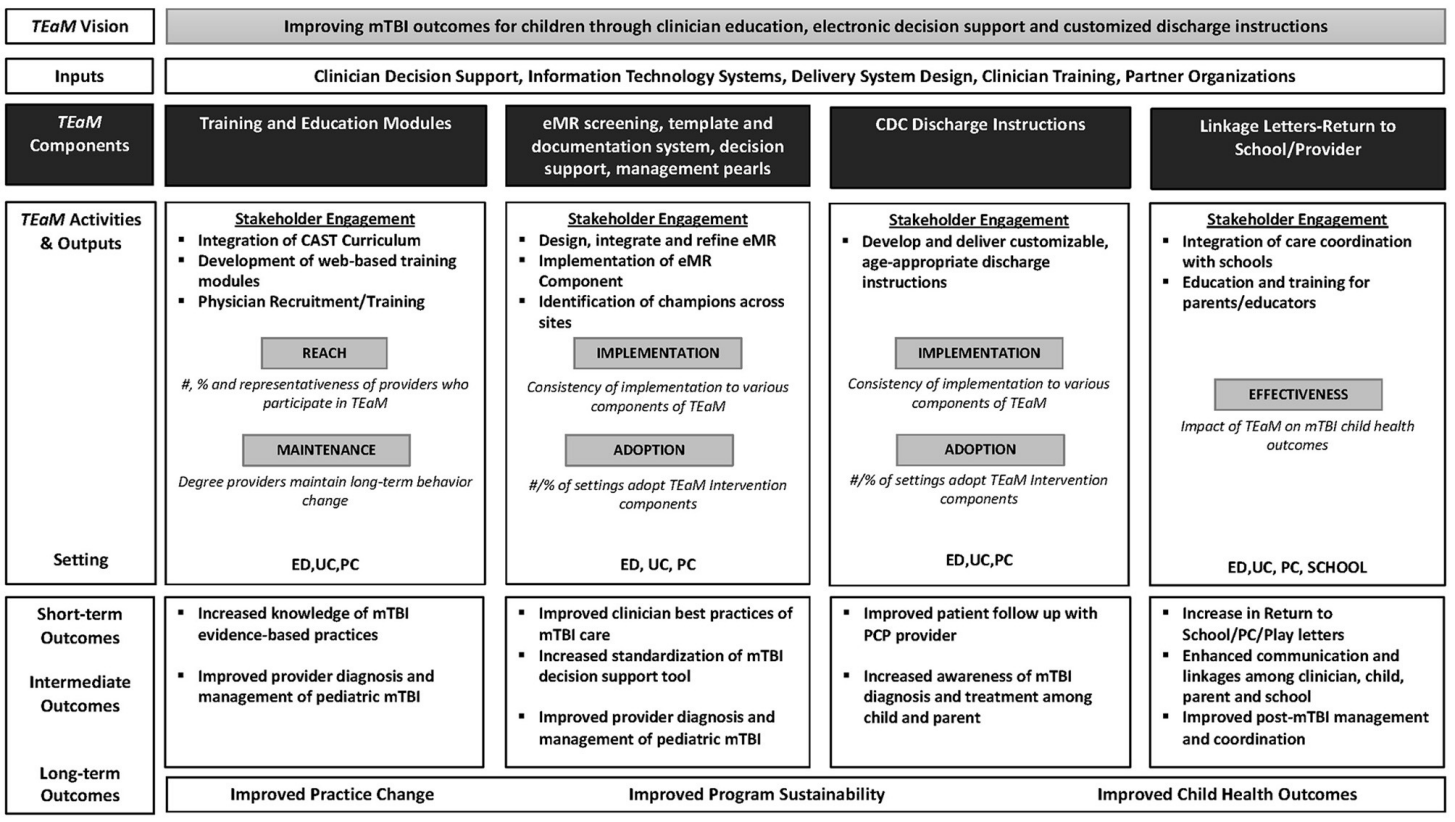
*TEaM* Logic Model inspired by and adapted from “Logic model for the BETTER 2 program” by Donna Patricia Manca et al. licensed under CC BY 4.0.

The emphasis on HCP education, training, and communication linkage tools addresses the research priority in mTBI. Furthermore, this model evaluates the sustainability of the *TEaM* Intervention following the funding period through ongoing engagement and collaboration of stakeholders within the healthcare and school systems, community partners, and funding agencies. This knowledge may assist researchers, funders, and policymakers in identifying specific outcome measures, contextual factors and, clinician practices surrounding pediatric mTBI. The *TEaM* Program Logic Model (Figure 2) was inspired by and adapted from the work of Manca et al. (57) to assist in program planning. The logic model describes the inputs, outputs/activities defining each applicable dimension of the RE-AIM framework, projected short, intermediate, and, long-term outcomes of the *TEaM* Intervention. The *TEaM* Intervention has the potential to provide a nationally recognized evidence-based intervention developed and guided by the best available evidence, ultimately promoting usability, feasibility, and scalability across organizational settings. The program evaluation component of the *TEaM* Intervention may provide the knowledge needed to better understand the application of a multi-component, multi-setting intervention into the real-world and increase the uptake and dissemination of pediatric mTBI guidelines.

## Project Status

**Figure 3:**
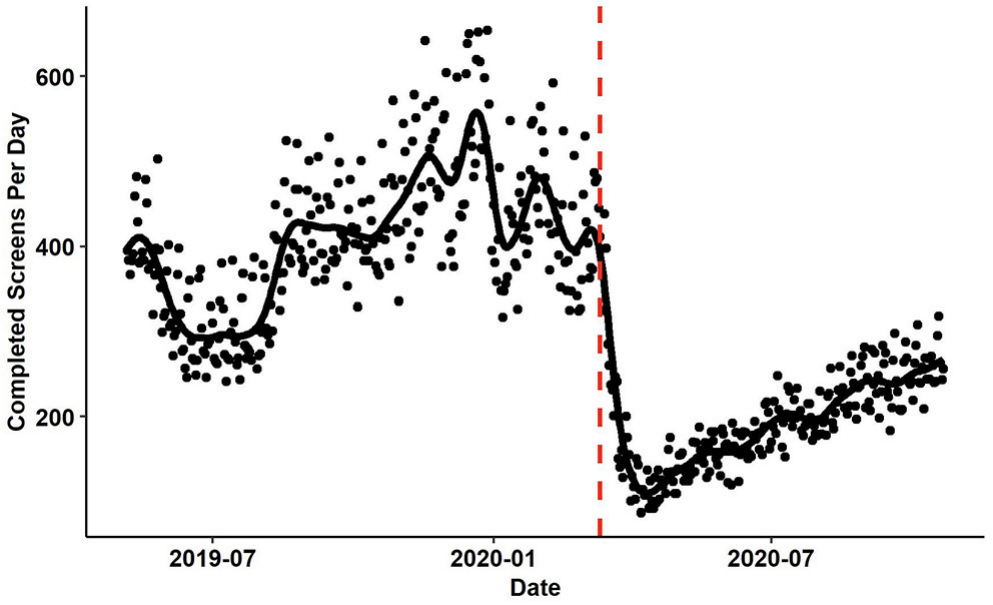
The number of patients screened per day at participating study sites. The red vertical line identifies March 11th, 2020 when the WHO declared the COVID-19 outbreak to be a pandemic. The black fit line was computed using smoothing splines. The λ parameter was set using generalized cross validation.

Study interventions requiring provider enrollment are in the development and refinement stages. Due to COVID-19, a delay within the study timeline has occurred as the investigative study staff as well as HCPs who will be randomized participating providers are Emergency Medicine providers or involved in programs that directly support that setting. This delay has impacted the expected timeline of provider recruitment. Similarly, patient presentations to the study sites initially decreased when distancing measures were first implemented in the United States (Figure 3). However, patient presentations have begun to return to pre-COVID-19 levels. Other project efforts such as publication and eMR/CDS refinement continue.

## Conclusion

Application of the RE-AIM framework (36) employing a mix-methods approach (RE-AIM QuEST) (38) is a model for evaluating the effectiveness of the *TEaM* Intervention to emphasize priorities of this public health concern surrounding pediatric mTBI. This evaluation method has the potential to provide the knowledge needed to critically appraise the impact of pediatric mTBI post- injury recovery interventions across multiple settings, enabling the uptake of best-available evidence within clinical practice.

## Ethics Statement

The study protocol was approved by the Institutional Review Board of Emory University (IRB Registration No. IRB00108674).

## Author Contributions

PT drafted the manuscript. TM wrote the data analysis. All authors contributed to the writing of the study protocol for grant funding, revised sections of the manuscript, and approved the final version of the manuscript.

## Funding

This study was funded by the Centers for Disease Control and Prevention (CDC), Grant Number 5U01CE002939-03.

## Conflict of Interest

The authors declare that the research was conducted in the absence of any commercial or financial relationships that could be construed as a potential conflict of interest.

## Publisher's Note

All claims expressed in this article are solely those of the authors and do not necessarily represent those of their affiliated organizations, or those of the publisher, the editors and the reviewers. Any product that may be evaluated in this article, or claim that may be made by its manufacturer, is not guaranteed or endorsed by the publisher.
